# The role of effect-based methods to address water quality monitoring in South Africa: a developing country’s struggle

**DOI:** 10.1007/s11356-022-23534-3

**Published:** 2022-10-14

**Authors:** Annika Kruger, Rialet Pieters, Suranie Horn, Catherina van Zijl, Natalie Aneck-Hahn

**Affiliations:** 1 Unit for Environmental Sciences and Management, North-West University, Potchefstroom, South Africa; 2 Occupational Hygiene and Health Research Initiative, North-West University, Potchefstroom, South Africa; 3 Environmental Chemical Pollution and Health Research Unit, University of Pretoria, Pretoria, South Africa

**Keywords:** Bioassay, Water quality management, Chemical compound mixtures, Water infrastructure, Analytical capacity

## Abstract

Water is an important resource, and it is a worldwide struggle to provide water of good quality to the whole population. Despite good governing laws and guidelines set in place to help protect the water resources and ensure it is of good quality for various consumers, the water quality in South Africa is worsening due to lack of management. The deteriorating infrastructure is becoming progressively worse, due to corruption and insufficient funds. The ever-increasing number of toxicants, as well as the identification of emerging chemicals of concern, are also challenges South Africa is facing. Chemical analysis cannot determine the total biological effect of a mixture of chemical compounds, but this shortcoming can be addressed by adding effect-based methods (EBMs) to water quality monitoring programmes. In this paper, the current status of water quality monitoring in South Africa is discussed, as well as the capacity of the country to add EBMs to its water quality monitoring programmes to protect and improve human and animal life.

**Graphical abstract:**

Created in Biorender.com

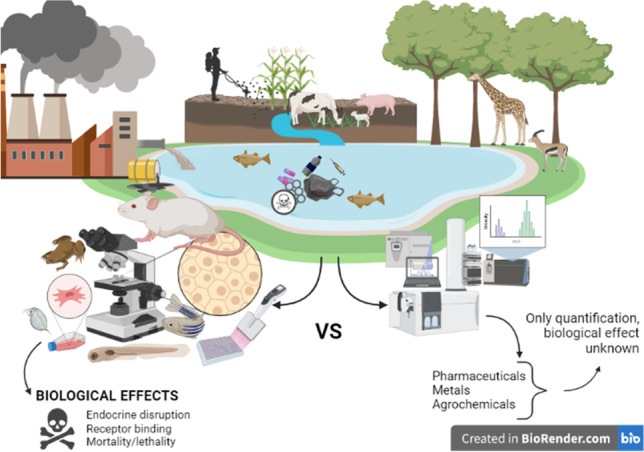

**Supplementary Information:**

The online version contains supplementary material available at 10.1007/s11356-022-23534-3.

## Introduction

The focus of this paper is water quality monitoring in South Africa, specifically that of chemical contaminants (not microbiological). Water quality guidelines for various water uses other than drinking water is outdated and little monitoring is done on a national scale. There are many and complex reasons why this is the situation, and they will be briefly discussed here. The main aim of this paper is to point out that, apart from management problems specifically in South Africa, there are also shortfalls globally in the traditional approach to monitoring the continuously growing list of toxicants in aquatic environments. The traditional approach refers to chemical instrumental analysis where targeted compounds are quantified. We will propose a viable solution to address this shortfall in the South African context.

## Status of compliance with water quality guidelines in South Africa (SA)

There are two laws that underpin South Africa’s water security: the Water Services Act (WSA) (Act 108 of 1997) and the National Water Act (NWA) (Act 36 of 1998). The WSA contains rules about how municipalities should provide potable water and sanitation services to households and other municipal water users. The NWA is the domain of the national government and contains rules about how the water in streams, rivers, dams and groundwater should be protected, used, developed, conserved, managed and controlled in an integrated manner (De la Harpe and Ramsden 2017).

Under the NWA, the national government is responsible for the establishment of the National Water Resource Strategy and one of the many issues that should be addressed is to set targets for water quality for different water resources. This was done in the form of the Water Quality Guidelines, but no updated guidelines, except for the drinking water guideline, had been published since 1996. The then Department of Water Affairs published eight volumes in a series of South African Water Quality Guidelines which had been updated in a second edition in 1996. Seven volumes contain water quality criteria—referred to as the Target Water Quality Range (TWQR)—together with other useful information. The eighth volume summarises the TWQR for each of the other volumes of different water uses: domestic, industrial, irrigation, livestock watering, aquaculture and aquatic ecosystem (supplementary Table 1). The domestic use water guideline had been fine-tuned into the South African National Standard 241 for drinking water (SANS 241 2015) and is the mandate of the WSA. The TWQR of the SANS 241 document is also included in the supplementary Table 1. It differs from that of domestic use and was mainly derived from the World Health Organization’s ‘Guidelines for drinking-water quality (SANS 241 2015). The SANS 241 is currently (2022) under revision again.

The collection of data and interpretation of information on water and sanitation are critical for effective water and sanitation management and this is done by the Directorate Resource Quality Information Services (RQIS) in the National Department of Water and Sanitation (DWS). They oversee several monitoring programmes on a national level: (i) chemical, (ii) microbial, (iii) eutrophication, (iv) toxicity, and (v) radioactivity monitoring programme along with (vi) ecosystem monitoring programme (DWS 2019a) in order to ensure good environmental water quality from which safe drinking water is prepared.

The National Toxicity Monitoring Programme (NTMP) is pertinent to this paper because it is concerned with only monitoring the concentrations of toxicants in the rivers and dams of South Africa with limited biological endpoints toxicity tests included. The latest reference regarding the work done by RQIS on the NTMP itself that could be found on the RQIS website is a ‘Draft phase 3: Pilot implementation and testing of design 2008–09’ in which a case study was reported: A number of sites were selected in the polluted Jukskei River in the Gauteng province. The aim was to establish the optimal sampling frequencies for various selected compounds which included several organochlorine pesticides, some alkyl phenols, a few phthalates, and toxaphene. A final version of this has not yet been published on the site. Some toxicity tests using *Danio rerio, Daphnia pulex, Poecilia reticulata, Selenastrum capricornutum* and engineered *Aliivibrio fischeri* enzyme inhibition tests were also included (DWS 2018). A shortlist of peer-reviewed research papers (Rimayi et al. 2015, 2016, 2017, 2018a, b, c) on a small number of sites for selected targeted compounds is also listed (DWS 2018). Rimayi co-authored more recent papers in the same vain (Batayi et al. 2020; Rimayi and Chimuka 2019; Rimayi et al. 2019) but these have not been referenced on the RQIS website.

The Blue Drop and the Green Drop Certification Programmes were introduced in 2008 and implemented in 2009 by DWS (Burges 2016). These programmes were incentive-based and aimed to improve drinking water quality (Blue Drop) and management of wastewater treatment plants (WWTPs) (Green Drop) which are the responsibilities of municipalities. One of the requirements of the Blue Drop programme is that the SANS 241 guidelines should be met. And one of the requirements for the Green Drop programme is that at a minimum, the general effluent standard (supplementary Table 1 ‘General effluent standard’) should be met in the case of an unlicenced WWTP (Government Gazette 2013). A licenced WWTP would receive its own customised requirements upon receiving its licence. The customisation is based on the size of the WWTP and the receiving river. In his 2020 State of the Nation Address, South African president Cyril Ramaphosa said the government is working to revive the Blue and Green Drop certification programme, which was disbanded in 2014 (Bega 2021), but the last Blue Drop and Green Drop report found on the Department of Water and Sanitation’s webpage is from 2012. Public interest organisations such as AfriForum took over the monitoring and tested 118 WWTPs and the drinking water quality of 220 towns (AfriForum 2020). Their report shows that 90 sewage systems and 5 towns did not comply with the limits of the general effluent standard (see supplementary Table 1) (AfriForum 2020).

Despite South Africa globally being hailed for its progressive water legislation (Takacs 2016), the implementation thereof, and specifically determining the quality of the water resources regularly had been slipping to a point where it seems to be non-existent, apart from a number of smaller research studies. This lack of performance and possible reasons had already been described succinctly by Schreiner (2013) in an opinion paper. Water quality monitoring is lacking due to corruption, lack of expertise, ineffective management of sewage and finances and as a result, there are gaps in the monitoring data (DWS 2019b). These gaps cause incomplete and erroneous assessments, that prevent decision-making.

Many people in rural communities in South Africa do not have access to piped water in their houses, let alone access to potable water of decent quality (Pearson et al. 2019). These communities use their nearest water source, often a river or dam, for inappropriate disposal of domestic waste and will use water from the same sources for household purposes (cooking, drinking, washing of clothes). Downstream users are at risk of various waterborne infections and diseases (Pearson et al. 2019). Two-thirds of South Africa is semi-arid with a variable annual rainfall which is far less than the global average (Blight and Fourie 2005). Almost half of South Africa receives less than 400 mm of rainfall per year (Schulze and Lynch 2006), and the mean annual runoff is 40 mm (Silberbauer 2020). Low rainfall results in freshwater being a scarce resource and with the population growing at a rapid rate, there is an increase in water use, which puts this resource under enormous pressure (Du Plessis 2019). The quality of freshwater is further decreasing due to anthropogenic activities such as mining, deforestation, urbanisation, agriculture, destruction of wetlands and river catchments (Pearson et al. 2019).

The sixth United Nations Sustainable Development Goal (SDG) requires that countries provide safe and affordable drinking water to everyone by 2030 (UNSDG 2015). According to Statistics SA (2018), the number of South Africans who have access to a source of drinking water (piped or tap water in the dwellings, off-site or on-site) in 2018 is 89%. South Africa therefore still has some way to go to meet the current SDG goals regarding water for domestic use.

## Compound mixtures in water

Although no country can analyse for all possible chemical contaminants, the list of chemicals proposed by regulatory guidelines (supplementary Table 1) to test for in South African waters are not as extensive as those of European and other developed countries. Furthermore, financial constraints leading to reduced capacity and skilled analysts in South Africa, prevent rigorous monitoring. In addition to the short list of chemicals that only includes metals and selected organic chemical pollutants (phenol, endosulfan, atrazine and trihalomethanes), we are also not monitoring the combined effects of the toxicants on a national scale.

By monitoring only a number of selected individual chemicals the potential harm posed by the chemical mixtures cannot be accurately assessed. Therefore, the probability of overlooking significant risks is high and increasing (Brack et al. 2019; De Baat et al. 2019). The solution would be to use integrative methods that would evaluate the possibility of complex mixtures causing harm. These complex mixtures may include compounds unbeknownst to be harmful, but because of the integrative approach their effects too will be quantified. Thus, the challenge is to characterize chemical pollution comprehensively, using limited resources, still diagnosing the impact of chemical pollution effectively. This needs to be done to prevent risks to ecosystems and human health, provide safe drinking water with limited treatment costs, and improve monitoring programmes (Brack et al. 2019; De Baat et al. 2019).

In a policy brief, SOLUTIONS (an European collaborative project) recommends integrating effect-based methods (EBMs) for diagnosis and monitoring of water quality (Brack et al. 2019). Effect-based methods are bioanalytical methods using the response of whole organisms (in vivo) or cellular bioassays (in vitro) to detect and quantify the effects of groups of chemicals on toxicological endpoints of concern. These assays should address both short-term toxicity (e.g. fish embryo vitality, algal growth) as well as include proxies for long-term effects (e.g. endocrine activity, mutagenicity, activated stress responses) (Brack et al. 2019). Despite the 45 priority substances identified by the European Union (2013) and approximately 300 River Basin-Specific Pollutants in different EU member states, it has been demonstrated that these substances reflect only a site-specific and typically unknown fraction of the overall chemical risk (Moschet et al. 2014). For this reason, Brack et al. (2019) suggested EBMs that would allow for detecting the combined effects of mixtures. This suggestion in fact, had been done in New Zealand, Australia, and Canada (Brunner et al. 2020; Kittinger et al. 2015).

## Effect-based methods as a potential solution

Effect-based methods (EBMs) reveal effects posed by chemical compound mixtures which have the same mode of action and are used to detect and quantify effects caused by these chemical mixtures (Brack et al. 2019; Könemann et al. 2018). König et al. (2017) evaluated the impact of untreated wastewater on the Danube River in Europe using both chemical instrumental anaysis and in vitro bioassays. A mass balance approach revealed a good agreement between the oestrogen, androgen, and glucocorticoid bioassays and the concentrations for the hormones. However, the chemical instrumental analysis explained less than 1% of the effects quantified by the bioassay for xenobiotic metabolism and 0–12% for adaptive stress responses. This study by König et al. (2017), and others such as Könemann et al., (2018), is a demonstration of the usefulness of EBMs complementing chemical instrumental analysis.

Brack et al. (2019) list five uses of EBMs: (i) detecting the effects of compound mixtures in water resources and demonstrating their potential to affect aquatic organisms and human health; (ii) minimising the risk to overlook harmful chemicals, metabolites and chemical mixtures; (iii) detecting hot spots of contamination for future monitoring; (iv) identifying the risk drivers and prioritising them for management measure; and (v) explaining how much of the ecological status is due to chemical pollution.

To support the River Basin Management Planning of Europe, Brack et al. (2019) suggested a battery of EBMs covering important ecotoxicological endpoints for pelagic communities, by using at least three tests: 96 h fish embryo acute toxicity, 48-h *Daphnia* sp. immobilisation, and 72-h inhibition of algae population growth. These are to be supplemented by in vitro assays that determine effects via specific modes of action (MoA) such as endocrine disruption, mutagenicity and activation of cellular defence mechanisms. They further give guidelines as to how the samples must be treated to ensure dose-relevant responses by the EBMs and the adoption of the regulatory frameworks to make use of EBMs to address currently established effects but also to tackle emerging endpoints of concern (Brack et al. 2019).

The South African government must meet the mandate of the WSA and NWA by better implementing the water quality monitoring programmes in existence in the country. But these programmes will benefit from supplementation by the EBM. A battery of appropriate EBMs should be validated for South African conditions. Due to environmental conditions in South Africa, different from that of the northern hemisphere, such as low precipitation, long daylight hours, and high UV radiation, lower levels of halogenated dioxin-like compounds are usually found in the water column. Selecting the AhR in vitro tests would therefore be useful for screening sediment (where these compounds are likely to accumulate), but not the water. The biota generally used in acute toxicity testing may be substituted with South African species.

There should also be cognisance of the role of sample processing: Extraction methods are optimised to ensure the best recoveries for specific target chemicals or groups of chemicals (Abbas et al. 2019). If these methods are applied to EBMs, which does not target specific chemicals but aims to assess the whole complex mixture, it may result in a misrepresentation of the actual activity of the sample. According to Abbas et al., (2019), sample preparation and extraction critically influenced the outcome of the various EBMs they tested: reporter gene EBMs for ER, AR, AhR, retinoic acid, retinoid X, vitamin D, thyroid receptor, the Ames fluctuation test, the umu test, and cytotoxicity. Extractions at pH 7 were most effective in recovering toxicity, but simultaneously masking the other endpoint under investigation. Lower pH extractions, e.g. pH 2.5 showed less cytotoxicity. Sample matrix may also affect the outcome of an EBM such as the co-extracted dissolved organic carbon that adsorps 17β-oestradiol in the antagonistic mode of the assay (Neale et al. 2015). Active compounds may be transformed by physicochemical and biological processes, modulating the biological effects under investigation.

## Effect-based methods available in South Africa

The general availability of EBMs in South Africa is investigated (Table 1) in order to get a sense of the capacity of the country to use effect-based screening of water quality supplemented by chemical instrumental analysis. An extensive literature search was done to determine the variety of EBMs available globally to compare to what is available in South Africa. Non-specific toxicity tests such as the ostracod and fish lethality assays are relatively common and well established for water licensing purposes (Government Gazette 1998). However, the lack of International Organization of Standardization (ISO) and Organisation of Economic Co-operation and Development (OECD) methods investigating mode-of-action effects such as endocrine disruption and dioxin-like activity in this section is an indication of the general shortfall that a country like South Africa experiences. As a starting point to address this shortfall, we provide an extensive list of methods already available in research laboratories but without standardised methods (supplementary Table 2). With this paper, we aim to motivate including internationally validated tests that investigate various biological endpoints (not only non-specific toxicity) to be developed and added to regulatory guidelines.

**Table 1:** Standardised tests available in South Africa

Assay/assay type	Endpoint	Biological agent	Type of water	Standard used	Reference
Algal growth inhibition assay	Growth inhibition	*Selenastrum capricornutum*	Carwash effluent	OECD Guideline 201:^1984^	Tekere et al. 2016
Algal growth inhibition assay	Growth inhibition	*Pseudokirchneriella subcapitata*		ISO 8692:^2012^	
Ames	Colony formation	*Salmonella typhimurium*	Freshwater	ISO 11350:^2012^	
Biotox assay	Bioluminescence inhibition	*Aliivibrio fischeri* (= *Vibrio fischeri*)	Wastewater effluent, Carwash effluent	ISO 11348–3:^2007^	Surujlal-Naicker et al. 2015, Tekere et al. 2016
*Daphnia/Ceriodaphnia* lethality test	Mortality	*Daphnia magna*	Carwash effluent	US EPA: 2002	Tekere et al. 2016
Fish lethality test	Mortality	*Poecilia reticulata,*	Carwash effluent	US EPA: 1996	Tekere et al. 2016
Ostracod toxkit-F	Growth inhibition	*Heterocypris incongruens*	River	ISO 14371:^2012^	Singh et al. 2017

## Acknowledging the challenges

There is room for both chemical instrumental analysis of compounds and EBMs in meeting water quality requirements: EBMs are crucial for determining total biological responses caused by toxicants to which an organism might be exposed to. Chemical instrumental analysis is useful for identifying the compounds responsible for these biological effects as well as monitoring for expected hazardous compounds. In a study by Vogt et al. (2019), dioxin-like responses was determined for sediment, using the H4IIE-*luc* assay. The assay’s responses were compared to the toxicity calculation based on the quantitative analysis of those compounds that could possibly be causing the biological response. The risk to sediment-dwelling organisms were greater according to the H4IIE-*luc* assay results than what could be predicted with the instrumental chemical analysis (Vogt et al. 2019). However, the only way of identifying the recalcitrant compounds was to use instrumental chemical analysis. In another study, the bioassay-equivalents also showed a greater risk than that determined from the concentration of the targeted chemicals (polycyclic aromatic hydrocarbons) (Pheiffer et al. 2019).

## Conclusion

South Africa, like many other countries, battles with the continued increase in the number of toxicants to monitor for in its water resources. Due to years of neglect and lack of maintenance, the country’s water infrastructure is also not functioning properly. This contributes to the battle of water quality monitoring, despite having the best laws governing this valuable natural resource. The application of EBMs to determine water quality together with the standard instrumental monitoring might not improve the failing infrastructure of South Africa. However, it may go a long way toward creating a holistic overview of water quality supporting the reinstatement of monitoring programmes left by the way-side. There are pockets of existing knowledge, especially in academic research fields based at universities where a spectrum of the necessary EBMs already exists.

If the Blue and Green Drop status programmes are to be reinstated, the country will also benefit from investing time, money and energy into establishing a battery of EBMs with which it may screen water quality. However, much time and scrutiny must be spent on selecting applicable assays for the different water uses, taking into consideration the level of training and laboratory infrastructure in the poorer municipalities and provinces. The necessary training should be made available to those institutions responsible for specifically potable water quality, to enable staff to conduct a variety of EBMs. It is not only the execution of the EBMs that should receive attention, but also the decision-making process upon which assays are selected. There is room and scope for in vivo and in vitro EBMs, for assays merely distinguishing between life and death and those designed to identify mode of action.

## Supplementary Information

Below is the link to the electronic supplementary material.

Supplementary file1 (DOCX 42.1 KB)

Supplementary file2 (DOCX 40.5 KB)

## Data Availability

Not applicable.
