# Supplementary material for: The role of effect-based methods to address water quality monitoring in South Africa: a developing country’s struggle
Source: Environ Sci Pollut Res Int. 2022 Oct 14;29(56):84049–55. doi: 10.1007/s11356-022-23534-3 (PMC9646548; doi:10.1007/s11356-022-23534-3)
Supplement: Supplementary file 1 — Supplementary file1 (DOCX 42.1 KB) [file 11356_2022_23534_MOESM1_ESM.docx]

Supplementary Table 1: Guidelines stipulated by the Department of Water Affairs and Forestry (DWAF) in 1996 and the South African National Standards (SANS) 241 in 2015

| Constituent in µg/L | DWAF V1 Domestic use | DWAF V2 Recreational use | DWAF V3 Industrial use | DWAF V4 Agricultural use: Irrigation | DWAF V5 Agricultural use: Livestock watering | DWAF V6 Agricultural use: Aquaculture | DWAF V7 Aquatic Ecosystems | SANS 241 | General effluent standard | |
| --- | --- | --- | --- | --- | --- | --- | --- | --- | --- | --- |
|  |  |  |  |  |  |  |  |  | General limit | Special limit |
| Alkalinity (CaCO_3_) |  |  | i) 0–5$\times$10^4^ |  |  | 2$\times$10^4^–10^5^ |  |  |  |  |
|  |  |  | ii) 0–1.2$\times$10^5^ |  |  |  |  |  |  |  |
|  |  |  | iii) 0–3$\times$10^5^ |  |  |  |  |  |  |  |
|  |  |  | iv) 0–10^6^ |  |  |  |  |  |  |  |
| Aluminium | 0–150 |  |  | 0–5$\times$10^3^ | 0–5$\times$10^3^ | < 30 | 5–10 | $\leq$ 300 |  |  |
| Ammonia | 0–1$\times$10^3^ |  |  |  |  | 0–25 | 0–7$\times$10^-9^ | $\leq$ 1.5$\times$10^3^ | 6$\times$10^3^ | 2$\times$10^3^ |
| Antimony |  |  |  |  |  |  |  | $\leq$ 20 |  |  |
| Arsenic | 0–1$\times$10^-8^ |  |  | 0–100 | 0–10^3^ | 0–50 | 10 | $\leq$ 10 | 20 | 0 |
| Atrazine | 0–2$\times$10^-9^ |  |  |  |  | 0–1.8$\times$10^-8^ | 10 |  |  |  |
| Barium |  |  |  |  |  |  |  | $\leq$ 700 |  |  |
| Beryllium |  |  |  | 0–100 |  |  |  |  |  |  |
| Boron |  |  |  | 0–500 | 0–5$\times$10^3^ |  |  | $\leq$ 2.4$\times$10^3^ | 10^3^ | 500 |
| Cadmium | 0–5 |  |  | 0–10 | 0–10 |  | 0–0.4 | $\leq$ 3 | 5 | 1 |
| Calcium | 0–3.2$\times$10^4^ |  |  |  | 0–10^6^ |  |  |  |  |  |
| Chemical oxygen demand |  |  | i) 0–10^4^ |  |  |  |  |  | 7.5$\times$10^4^ | 3$\times$10^4^ |
|  |  |  | ii) 0–1.5$\times$10^4^ |  |  |  |  |  |  |  |
|  |  |  | iii) 0–3$\times$10^4^ |  |  |  |  |  |  |  |
|  |  |  | iv) 0–7.5$\times$10^4^ |  |  |  |  |  |  |  |
| Chloride |  |  | i) 0–2$\times$10^4^ | 0–10^5^ | 0–1.5$\times$10^6^ | < 6$\times$10^5^ |  | $\leq$ 3$\times$10^5^ |  |  |
|  |  |  | ii) 0–4.5$\times$10^4^ |  |  |  |  |  |  |  |
|  |  |  | iii) 0–10^5^ |  |  |  |  |  |  |  |
|  |  |  | iv) 0–10^5^ |  |  |  |  |  |  |  |
| Chlorine | 0–10^5^ |  |  |  |  |  | 0.2 | $\leq$ 5$\times$10^3^ | 250 | 0 |
| Chromium (VI) |  |  |  | 0–100 | 0–10^3^ | < 0–2$\times$10^-8^ | 7 |  | 50 | 20 |
| Cobalt |  |  |  | 0–50 | 0–10^3^ |  | 0–1.4 |  |  |  |
| Copper | 0–10^3^ |  |  | 0–200 | 0–500 | < 5 |  | $\leq$ 2$\times$10^3^ | 10 | 2 |
| Cyanide |  |  |  |  |  | < 20 | 1 | $\leq$ 200 | 20 | 10 |
| Dissolved organic carbon | 0–5$\times$10^3^ |  |  |  |  |  |  |  |  |  |
| Dissolved oxygen |  |  |  |  |  | 6–9 | 80–120% of saturation |  |  |  |
| Endosulfan |  |  |  |  |  |  | 0.01 |  |  |  |
| Fluoride | 0–10^3^ |  |  | 0–2$\times$10^3^ | 0–2$\times$10^3^ |  | 750 | $\leq$ 2.5$\times$10^5^ | 10^3^ | 10^3^ |
| Iron | 0–100 |  | i) 0–100 | 0–5$\times$10^3^ | 0–10^4^ | < 10 | < 10% of background dissolved iron concentration | $\leq$ 2$\times$10^3^ | 300 | 300 |
|  |  |  | ii) 0–200 |  |  |  |  |  |  |  |
|  |  |  | iii) 0–300 |  |  |  |  |  |  |  |
|  |  |  | iv) 0–10^4^ |  |  |  |  |  |  |  |
| Lead | 0–10^-8^ |  |  | 0–200 | 0–100 | < 10 | 0–1.2 | $\leq$ 10 | 10 | 6 |
| Lithium |  |  |  | 0–2.5$\times$10^3^ |  |  |  |  |  |  |
| Magnesium | 0–30 000 |  |  |  | 0–5$\times$10^5^ |  |  |  |  |  |
| Manganese | 0–50 |  | i) 0–50 | 0–20 | 0–10^4^ | < 100 | 180 | $\leq$ 400 | 100 | 100 |
|  |  |  | ii) 0–100 |  |  |  |  |  |  |  |
|  |  |  | iii) 0–200 |  |  |  |  |  |  |  |
|  |  |  | iv) 0–1$\times$10^4^ |  |  |  |  |  |  |  |
| Mercury | 0–10^-9^ |  |  |  | 0–10^3^ | < 10^3^ | 0.04$\times$10^-9^ | $\leq$ 6 | 5 | 1 |
| Molybdenum |  |  |  | 0–10 | 0–10 |  |  |  |  |  |
| Monochloramine |  |  |  |  |  |  |  | $\leq$ 3$\times$10^3^ |  |  |
| Nickel |  |  |  | 0–200 | 0–10^3^ |  |  | $\leq$ 70 |  |  |
| Nitrate | 0–6$\times$10^3^ |  |  |  | 0–10^5^ | < 3$\times$10^5^ |  | $\leq$ 1.1$\times$10^4^ | 1.5$\times$10^4^ | 1.5$\times$10^3^ |
| Nitrite |  |  |  |  |  | < 50 |  | $\leq$ 900 |  |  |
| Nitrogen (inorganic) |  |  |  | 0–5$\times$10^3^ |  |  | v) < 500 |  |  |  |
|  |  |  |  |  |  |  | vi) 500–2.5$\times$10^3^ |  |  |  |
|  |  |  |  |  |  |  | vii) 2.5$\times$10^3^–10^4^ |  |  |  |
|  |  |  |  |  |  |  | viii) >10^4^ |  |  |  |
| pH | 6–9 | 6.5–8.5 | i) 7–8 | 6.5–8.4 |  | 6.5–9 | 5–10 | $\geq$ 5–$\leq$ 9.7 | 5.5–9.5 | 5.5–7.5 |
|  |  |  | ii) 6.5–8 |  |  |  |  |  |  |  |
|  |  |  | iii) 6.5–8 |  |  |  |  |  |  |  |
|  |  |  | iv) 5–10 |  |  |  |  |  |  |  |
| Phenol | 0–10^-9^ |  |  |  |  | < 10^3^ |  | $\leq$ 10 |  |  |
| Phosphorus (inorganic) |  |  |  |  |  | 100 | v) < 5$\times$10^-9^ |  | 10^4^ | 10^3^ (median); 2.5$\times$10^3^ (max) |
|  |  |  |  |  |  |  | vi) 5$\times$10^-9^–2 5$\times$10^-9^ |  |  |  |
|  |  |  |  |  |  |  | vii) 2 5$\times$10^-9^ –250$\times$10^-9^ |  |  |  |
|  |  |  |  |  |  |  | viii) >250$\times$10^-9^ |  |  |  |
| Potassium | 0–5$\times$10^4^ |  |  |  |  |  |  |  |  |  |
| Selenium | 0–2$\times$10^-8^ |  |  | 0–20 | 0–5$\times$10^-8^ | < 300 | 2 | $\leq$ 40 | 20 | 20 |
| Silica |  |  | i) 0–5$\times$10^3^ |  |  |  |  |  |  |  |
|  |  |  | ii) 0–10^4^ |  |  |  |  |  |  |  |
|  |  |  | iii) 0–2$\times$10^4^ |  |  |  |  |  |  |  |
|  |  |  | iv) 0–1.5$\times$10^5^ |  |  |  |  |  |  |  |
| Sodium adsorption ratio |  |  |  | 2 |  |  |  |  |  |  |
| Sodium | 0–10^5^ |  |  | 0–7$\times$10^4^ | 0–2$\times$10^6^ |  |  | $\leq$ 2$\times$10^5^ |  |  |
| Sulphate | 0–2$\times$10^5^ |  | i) 0–3$\times$10^4^ |  | 0–10^6^ |  |  | $\leq$ 5$\times$10^5^ |  |  |
|  |  |  | ii) 0–8$\times$10^4^ |  |  |  |  |  |  |  |
|  |  |  | iii) 0–2$\times$10^5^ |  |  |  |  |  |  |  |
|  |  |  | iv) 0–5$\times$10^5^ |  |  |  |  |  |  |  |
| Sulphide |  |  |  |  |  | < 1 |  |  |  |  |
| Suspended solids |  |  | i) 0–3$\times$10^3^ | 0–5$\times$10^4^ |  |  |  |  | 2.5$\times$10^4^ | 10^4^ |
|  |  |  | ii) 0–5$\times$10^3^ |  |  |  |  |  |  |  |
|  |  |  | iii) 0–5$\times$10^3^ |  |  |  |  |  |  |  |
|  |  |  | iv) 0–2.5$\times$10^4^ |  |  |  |  |  |  |  |
| Total chromium |  |  |  |  |  |  |  | $\leq$ 50 |  |  |
| Total dissolved solids | 0–4.5$\times$10^5^ |  | i) 0–10^5^ | 0–256 | 0–10^6^ | < 2$\times$10^3^ |  |  |  |  |
|  |  |  | ii) 0–10^5^ |  |  |  |  |  |  |  |
|  |  |  | iii) 0–4.5$\times$10^5^ |  |  |  |  |  |  |  |
|  |  |  | iv) 0–1.6$\times$10^6^ |  |  |  |  |  |  |  |
| Total Hardness |  |  | i) 0–5$\times$10^4^ |  |  | 20–100 |  |  |  |  |
|  |  |  | ii) 0–10^5^ |  |  |  |  |  |  |  |
|  |  |  | iii) 0–2.5$\times$10^5^ |  |  |  |  |  |  |  |
|  |  |  | iv) 0–10^6^ |  |  |  |  |  |  |  |
| Total organic carbon |  |  |  |  |  |  |  | $\leq$10 |  |  |
| Trihalomethanes | 0–10^-7^ |  |  |  |  |  |  | $\leq$ 1 |  |  |
| Uranium | 0–70 |  |  | 0–10 |  |  |  | $\leq$ 10 |  |  |
| Vanadium | 0–100 |  |  | 0–100 | 0–10^3^ |  |  |  |  |  |
| Zinc | 0–3$\times$10^3^ |  |  | 0–10^3^ | 0–2$\times$10^4^ |  | 2 | $\leq$ 5$\times$10^3^ | 100 | 40 |
| References | DWAF, 1996a | DWAF, 1996b | DWAF, 1996c | DWAF, 1996d | DWAF, 1996e | DWAF, 1996f | DWAF, 1996g | SANS 241, 2015 | Government Gazette, 2013 | |

^i: Industrial processes that needs high quality water; ii: Water of intermediate to high quality is required for these processes; iii: Domestic water quality with baseline minimum standards; iv: Industrial processes which can use water of any quality; v: Under oligotrophic conditions; vi: Mesotrophic conditions; vii: Eutrophic conditions; viii: Hypertrophic conditions^

References

DWAF, Department of Water Affairs and Forestry (1996a) South African Water Quality Guidelines (second edition). Volume 1: Domestic Use

DWAF, Department of Water Affairs and Forestry (1996b) South African Water Quality Guidelines for Coastal Marine Waters. Volume 2: Recreational Use

DWAF, Department of Water Affairs and Forestry (1996c) South African Water Quality Guidelines (second edition). Volume 3: Industrial Use

DWAF, Department of Water Affairs and Forestry (1996d) South African Water Quality Guidelines (second edition). Volume 4: Agricultural Use: Irrigation

DWAF, Department of Water Affairs and Forestry (1996e) South African Water Quality Guidelines (second edition). Volume 5: Agricultural Use: Livestock Watering

DWAF, Department of Water Affairs and Forestry (1996f) South African Water Quality Guidelines (second edition). Volume 6: Agricultural Use: Aquaculture

DWAF, Department of Water Affairs and Forestry (1996g) South African Water Quality Guidelines. Volume 7: Aquatic Ecosystems

Government Gazette (2013) Vol 579 no 3682 Government Notice 665 National Water Act (36/1998): revision of general authorisation in terms of section 39 of the Act. https://archive.opengazettes.org.za/archive/ZA/2013/government-gazette-ZA-vol-579-no-36820-dated-2013-09-06.pdf Date of access: 18 February 2022

SANS, South African National Standards 241-1 (2015) Drinking water. Part 1: microbiological, physical, aesthetic and chemical determinands. 2nd ed. Pretoria. SABS
