# Supplementary material for: The role of effect-based methods to address water quality monitoring in South Africa: a developing country’s struggle
Source: Environ Sci Pollut Res Int. 2022 Oct 14;29(56):84049–55. doi: 10.1007/s11356-022-23534-3 (PMC9646548; doi:10.1007/s11356-022-23534-3)
Supplement: Supplementary file 2 — Supplementary file2 (DOCX 40.5 KB) [file 11356_2022_23534_MOESM2_ESM.docx]

Supplementary Table 2: *In vivo* and *in vitro* analyses performed in South Africa to test water quality

| **Target mode of action** | **No.** | ***In vivo/ in vitro*** | **Assay/Assay type** | **Endpoint** | **Biological agent** | | ***Type of water** | **Reference** |
| --- | --- | --- | --- | --- | --- | --- | --- | --- |
| **Non-specific toxicity (baseline toxicity)** | | | | | | | | |
| Toxicity | 1 | *In vitro* | Biotox assay | Bioluminescence inhibition | *Aliivibrio fischeri* (=*Vibrio fischeri*) | | Wastewater effluent  Carwash effluent | Surujlal-Naicker et al., 2015, Tekere et al., 2016 |
|  | 2 | *In vitro* | Mammalian cell colony formation inhibition test | Colony formation | Buffalo Green monkey (BGM) kidney cells and Chinese hamster V79 cells | | Freshwater | Slabbert et al., 1998 |
|  | 3 | *In vitro* | Bacterial growth inhibition assay | Growth inhibition | *Pseudomonas putida* | | Freshwater | Slabbert et al., 1998 |
|  | 4 | *In vivo* | Ostracod toxkit-F | Growth inhibition | *Heterocypris incongruens* | | River | Singh et al., 2017 |
|  | 5 | *In vivo* | Microplate-based *Hydra attenuata* assay | Growth rate | *Hydra vulgaris* (=*Hydra attenuata*) | | Wetland, lake, treated sewage and creek | Oberholster et al., 2008 |
|  | 6 | *In vivo* | Chronic acid tolerance bioassay | Growth rate (mass & length) | *Amietophrynus maculatus, Chiromantis xerampelina, Hildebrandtia ornata*, *Pyxicephalus edulis* | | Rain water | Farquharson et al., 2016 |
|  | 7 | *In vivo* |  | Hatching rate | *A. maculatus, C. xerampelina, H. ornata*, *P. edulis* | | Rain water | Farquharson et al., 2016 |
|  | 8 | *In vivo* |  | Hatching rate | *Pyxicephalus adspersus* | | Wetland, lake, treated sewage and creek | Oberholster et al., 2008 |
|  | 9 | *In vivo* | Diptera assay | Mortality | *Chironomus caffrarius* | | River | Singh et al., 2017 |
|  | 10 | *In vivo* | Fish lethality test | Mortality | *Danio rerio, Labeobarbus aeneus, Oreochromis mossambicus, Poecilia reticulata, Pseudocrenilabrus philander, Tilapia sparrmanii* | | Freshwater, Carwash effluent, | Botha et al., 2015, Brand et al., 2020, Slabbert et al., 1998, Tekere et al., 2016 |
|  | 11 | *In vivo* | *Daphnia/Ceriodaphnia* lethality test | Mortality | *Daphnia magna Daphnia pulex*  *Ceriodaphnia dubia* | | Freshwater, Wetland, lake, treated sewage and creek, Carwash effluent | Botha et al., 2015, Oberholster et al., 2008, Slabbert et al., 1998, Tekere et al., 2016 |
|  | 12 | *In vivo* |  | Swimming behaviour | *D. rerio* | | Freshwater | Brand et al., 2020 |
| Staining assays | 13 | *In vitro* | Resazurin cell proliferation assay | Mitochondrion activity | GH3 rat pituitary carcinoma cells | | Drinking water, surface and treated wastewater, drinking water treatment plant | Simba, 2017 |
|  | 14 | *In vitro* | MTT viability assay | Mitochondrion activity | HuTu-80 human duodenum adenocarcinoma cell  H4IIE-*luc* rat hepatoma cell  MDA-kb2 human breast carcinoma cells | | Dam, River | Prinsloo et al., 2013  Pheiffer et al., 2019, Vogt et al., 2019  Powrie, 2016 |
|  | 15 | *In vitro* | WST-1 viability assay | Mitochondrion activity | RAW264.7 mouse macrophage cells | | Treated sewage, wastewater | Makene and Pool, 2015, Makene et al., 2016 |
|  | 16 | *In vitro* | XTT viability assay | Mitochondrion activity | RAW264.7 mouse macrophage cells | | Treated sewage | Makene and Pool, 2015 |
| **Specific toxicity** | | | | | | | | |
| Endocrine disruptioN | | | | | | | | |
| *Androgen receptor (AR)* | | | | | | | | |
| AR(ant)-agonism | 17 | *In vitro* | Reporter gene assay | Receptor mediated effects | MDA-kb2 human breast carcinoma cells | | River | De Jager et al., 2011, Powrie, 2016 |
|  | 18 | *In vitro* | YAS | Receptor mediated effects | *Saccharomyces cerevisiae* | | River | Truter et al., 2016 |
| *Oestrogen receptor (ER)* | | | | | | | | |
| ER(ant)-agonism | 19 | *In vitro* | E-screen (modified) | Proliferation | MCF-7 human breast carcinoma cells | | River | Swart et al., 2011 |
|  | 20 | *In vitro* | YES | Receptor mediated effects | *S. cerevisiae* | | Dam, Laboratory water, Treated drinking water, Wetland, Stream, wastewater, River, drinking water treatment plant | Aneck-Hahn et al., 2005, Aneck-Hahn et al., 2008, Aneck-Hahn et al., 2009, Archer et al., 2020, Du Preez and Slabbert, 2008, Patrick et al., 2020, Van Zijl et al., 2017, |
|  | 21 | *In vitro* | Reporter gene assay | Receptor mediated effects | T47D-kB*luc* human breast carcinoma cells | | Feedlot runoff, Treated drinking water | Patrick et al., 2020, Van Zijl et al., 2017 |
| Alternative ER techniques | 22 | *In vitro* | MCF-7 ERα ELISA | ERα protein concentration | MCF-7 breast carcinoma cells | | River | Swart and Pool, 2009, Swart et al., 2011 |
|  | 23 | *In vivo* | Gonadosomatic index  Urogenital papilla length index | Gonad size;  Urogenital papilla length | *Clarias gariepinus* | | Dam | Kruger et al., 2013 |
|  | 24 | *In vivo* | Vtg ELISA | Vitellogenin production | *D. rerio, O. mosambicus* | | Feedlot runoff, River | Du Preez and Slabbert, 2008, Swart et al., 2011 |
|  | 25 | *In vivo* | Primary rainbow trout hepatocyte assay | Vitellogenin (Vtg) production | *Oncorhynchus mykiss* | | Feedlot runoff | Du Preez and Slabbert, 2008 |
|  | 26 | *In vivo* | Vtg ELISA | Vitellogenin production | *Xenopus laevis* | | River, wastewater treatment plant | Pool, 2008 |
| *Thyroid receptor (TR)* | | | | | | | | |
| TR activity | 27 | *In vitro* | Reporter gene assay | Receptor mediated effects | GH3.TRE.*luc* rat pituitary tumour cells | | Drinking water, surface and treated wastewater, drinking water treatment plant | Simba, 2017 |
| DIOXIN-LIKE ACTIVITY | | | | | | | | |
| *Aryl hydrocarbon receptor (AhR)* | | | | | | | | |
| AhR activity | 28 | *In vitro* | Reporter gene assay | Receptor mediated effects | H4IIE-*luc* rat hepatoma cell | | Dam, River | Pheiffer et al., 2019, Vogt et al., 2019 |
| DEVELOPMENTAL TOXICITY | | | | | | | | |
|  | 29 | *In vivo* | Toad embryo teratogenicity test | Embryo development (size & length), pigmentation, head shape, form of spines and tails | *X. laevis* | | Freshwater | Slabbert et al., 1998 |
|  | 30 | *In vivo* | Abalone embryo development test | Operculate veliger stage embryo | *Haliotis midae* | | Industrial and domestic effluent, coastal waters | Shackleton et al., 2002 |
|  | 31 | *In vivo* | Invertebrate reproduction test | Reproduction | *D. magna, C. dubia* | | Freshwater | Slabbert et al., 1998 |
| HEPATOTOXICITY | | | | | | | | |
| Cytochrome P450 | 32 | *In vivo* | Demethylating fluorescent activity kit | Cytochrome P450 activity | *Caridina nilotica, C. gariepinus, Perna perna* | | River | Coetzee, 2015, Pheiffer, 2017, Van Rensburg et al., 2020 |
| METAL TOXICITY | | | | | | | | |
|  | 33 | *In vivo* | Metallothionein content | Metallothionein content | *Atractolytocestus huronensis, C. nilotica, C. gariepinus, Contracaecum sp., Cyprinus carpio,* *Dreissena polymorpha* | | Dam, River, Freshwater | Brand et al., 2019, Erasmus et al., 2020, Van Rensburg et al., 2020 |
| NEUROTOXICITY | | | | | | | | |
| Acetylcholinesterase (AChE) activity | 34 | *In vivo* | AChE activity assay | AChE activity | *A. huronensis, C. nilotica, C. gariepinus, Contracaecum sp., C. carpio, P. perna, T. sparmanii* | | River | Coetzee, 2015, Erasmus et al., 2020, Malherbe, 2013, Pheiffer, 2017, Van Rensburg et al., 2020 |
| IMMUNOTOXICITY | | | | | | | | |
| Cytotoxicity | 35 | *In vitro* | ELISA | LDH concentration | Whole blood culture | | River | Pool and Magcwebeba, 2009 |
| Immunity (cell mediated) | 36 | *In vitro* | ELISA | IFN-y concentration | Whole blood culture | | River | Pool and Magcwebeba, 2009 |
| Immunity (hormonal) | 37 | *In vitro* | ELISA | IL-10 concentration | Whole blood culture | | River | Pool and Magcwebeba, 2009 |
| Inflammatory activity | 38 | *In vitro* | ELISA | IL-6 concentration | RAW264.7 mouse macrophage cells  Whole blood culture | | River, Treated sewage, wastewater | Makene and Pool, 2015, Makene et al., 2016, Pool and Magcwebeba, 2009, Pool et al., 2000 |
|  | 39 | *In vitro* | Griess reaction | Nitric oxide concentration | RAW264.7 mouse macrophage cells | | Treated sewage, wastewater | Makene and Pool, 2015, Makene et al., 2016 |
| **Reactive toxicity** | | | | | |  |  |  |
| *Mutagenicity* | | | | | | | | |
|  | 40 | *In vitro* | Ames | Colony formation | *Salmonella typhimurium* | | Freshwater | Slabbert et al., 1998 |
|  | 41 | *In vitro* | Cell transformation test | Evidence of malignancies | Hamster embryo cells | |  |  |
| *Oxidative stress* | | | | | | | | |
|  | 42 | *In vivo* | Cellular energy allocation | Energy consumption (energy needed to reduce oxygen) | *A. huronensis, C. nilotica, C. gariepinus, Contracaecum sp., C. carpio, T. sparmanii* | | River | Erasmus et al., 2020, Malherbe, 2013, Pheiffer, 2017, Van Rensburg et al., 2019 |
|  | 43 | *In vivo* | Catalase | Enzyme activity | *A. huronensis, C. nilotica, C. gariepinus, Contracaecum sp., C. carpio, D. polymorpha, H. vittatus,* *P. perna, T. sparmanii* | | Freshwater, River | Brand et al., 2019, Coetzee, 2015, Erasmus et al., 2020, Gerber et al., 2018, Malherbe, 2013, Pheiffer, 2017, Van Rensburg et al., 2019 |
|  | 44 | *In vivo* | Superoxide dismutase | Enzyme activity | *A. huronensis, C. nilotica, C. gariepinus, Contracaecum sp., C. carpio, H. vittatus, P. perna* | | River | Coetzee, 2015, Erasmus et al., 2020, Gerber et al., 2018, Pheiffer, 2017, Van Rensburg et al., 2019 |
|  | 45 | *In vivo* | Glutathione-S-transferase | Enzyme activity | *D. polymorpha* | | Freshwater | Brand et al., 2019 |
|  | 46 | *In vivo* | Reduced glutathione | Glutathione content | *A. huronensis, C. nilotica, C. gariepinus, Contracaecum sp., C. carpio, H. vittatus* | | River | Erasmus et al., 2020, Gerber et al., 2018, Van Rensburg et al., 2019 |
|  | 47 | *In vivo* | Lipid peroxidation | Malondialdehyde production | *A. huronensis, C. nilotica, C. gariepinus, Contracaecum sp., C. carpio, D. polymorpha, H. vittatus, P. perna, T. sparmanii* | | River | Brand et al., 2019, Coetzee, 2015, Erasmus et al., 2020, Gerber et al., 2018, Malherbe, 2013, Pheiffer, 2017, Van Rensburg et al., 2019 |
|  | 48 | *In vivo* | Protein Carbonyl levels | Protein concentration | *A. huronensis, C. nilotica, C. gariepinus, Contracaecum sp., C. carpio, H. vittatus, P. perna, T. sparmanii* | | River | Coetzee, 2015, Erasmus et al., 2020, Gerber et al., 2018, Malherbe, 2013, Pheiffer, 2017, Van Rensburg et al., 2019 |
| **Low-complexity *in vivo* plant assays** | | | | | | | | |
| Algal growth and PSII inhibition | 49 | *In vivo* | Algal growth inhibition assay | Growth inhibition | *Selenastrum capricornutum* | | Carwash effluent, Freshwater | Slabbert et al., 1998, Tekere et al., 2016 |
| Cytotoxicity, growth inhibition | 50 | *In vivo* | Root growth | Root growth | *Allium cepa* | | Wetland, lake, treated sewage and creek | Oberholster et al., 2008 |
| Seed germination and root growth | 51 | *In vivo* | Seed germination | Seed germination | *Lactuca sativa* | | Wetland, lake, treated sewage and creek | Oberholster et al., 2008 |
|  | 52 | *In vivo* | Phytotoxkit-F | Seed germination, root and shoot growth inhibition | *Lepidium sativum, Sinapis alba, Sorghum saccharatum* | | River | Singh et al., 2017 |
| **Low complexity *in vivo* protozoan assay** | | | | | | | | |
| Oxygen uptake | 53 | *In vivo* | Protozoan oxygen uptake assay | Oxygen consumption rate | *Tetrahymena pyriformis* | | Freshwater, River | Bulannga and Schmidt 2022, Slabbert et al., 1998 |
| **Biomonitoring** | | | | | | | | |
|  | 54 | *In vivo* | Biological diatom index, Generic diatom index, Specific pollution index | Abundance and species richness | Diatoms | | River | Holmes and Taylor, 2015, Kock et al., 2019, Schoeman, 1979, Taylor et al., 2007 |
|  | 55 | *In vivo* | Fish diversity type indices, eg. Fish Response Assessment Index (FRAI) & Fish Assemblage Integrity Index (FAII) | Abundance and species richness | Fish | | River | Kleynhans 1999, Malherbe et al., 2015, Malherbe et al., 2016 |
|  | 56 | *In vivo* | South African Scoring System version 5 (SASS5) | Abundance and species richness | Macroinvertebrates | | River | De Necker et al., 2016, Malherbe, 2013, Malherbe et al., 2015, Malherbe et al., 2018, Van Deventer et al., 2021 |
|  | 57 | *In vivo* | Fish Health Assessment Index (FHAI) | Fish health | Fish | | Dam, River | Erasmus et al., 2019, Malherbe, 2013, Nibamureke et al., 2016, Sara et al., 2014, Wagenaar and Barnhoorn 2018, Wepener et al., 2011 |

References

Aneck-Hahn NH, De Jager C, Bornman MS, Du Toit D (2005) Oestrogenic activity using a recombinant yeast screen assay

(RCBA) in South African laboratory water sources. Water SA 31(2):253–256. https://doi.org/10.4314/wsa.v31i2.5192

Aneck-Hahn NH, Bornman MS, De Jager C (2008) Preliminary assessment of oestrogenic activity in water sources in Rietvlei

Nature Reserve, Gauteng, South Africa. Afr J Aquat Sci 33(3):249–254. https://doi.org/10.2989/AJAS.2008.33.3.7.619

Aneck-Hahn NH, Bornman MS, De Jager C (2009) Oestrogenic activity in drinking waters from a rural area in the Waterberg

District, Limpopo Province, South Africa. Water SA 35(3):245–251. https://doi.org/10.4314/wsa.v35i3.76760

Archer E, Wolfaardt GM, van Wyk JH, van Blerk N (2020) Investigating (anti) oestrogenic activities within South African

wastewater and receiving surface waters: implication for reliable monitoring. Environ Pollut 263:114424.

https://doi.org/10.1016/j.envpol.2020.114424

Bandelj E, van den Heuvel MR, Leusch FDL, Shannon N, Taylor S, McCarthy LH (2006) Determination of the androgenic

potency of whole effluents using mosquitofish and trout bioassays. Aquat Toxicol 80(3):237–248.

<https://doi.org/10.1016/j.aquatox.2006.08.011>

Botha TL, James TE, Wepener V (2015) Comparative aquatic toxicity of gold nanoparticles and ionic gold using a species sensitivity distribution approach. J Nanomater 2015.https://doi.org/10.1155/2015/986902

Brand SJ, Erasmus JH, Labuschagne M, Grabner D, Nachev M, Zimmermann S, Wepener V, Smit N, Sures B (2019)

Bioaccumulation and metal-associated biomarker responses in a freshwater mussel, Dreissena polymorpha, following short-term

platinum exposure. Environ Pollut 246:69–78. https://doi.org/10.1016/j.envpol.2018.11.061

Brand SJ, Botha TL, Wepener V (2020) Behavioural response as a reliable measure of acute nanomaterial toxicity in zebrafish

larvae exposed to a carbon-based versus a metal-based nanomaterial. Afr Zool 55(1):57–66.

<https://doi.org/10.1080/15627020.2019.1702098>

Bulannga RB, Schmidt S (2022) Uptake and accumulation of microplastic particles by two freshwater ciliates isolated from a local river in South Africa. Environ Res 204:112123. <https://doi.org/10.1016/j.envres.2021.112123>

Coetzee AE (2015) The assessment of organic pollutant exposure and effects along the KwaZulu-Natal coastline. Doctoral thesis, North-West University (South Africa), Potchefstroom Campus

De Necker L, Ferreira M, van Vuren JHJ, Malherbe W (2016) Aquatic invertebrate community structure of selected endorheic wetlands (pans) in South Africa. Inland Waters 6(3):303–313. https://doi.org/10.1080/IW-6.3.783

De Jager C, Swemmer A, Aneck-Hahn NH, Van Zijl C, Van Wyk S, Bornman MS, Barnhoorn IEJ, Jonker M, Van Vuuren JHJ, Burger AEC (2011) Endocrine disrupting chemical (EDC) activity and health effects of identified veterinary growth stimulants in surface and groundwater. Water Research Commission. Report No 1686/1/11

Erasmus JH, Malherbe W, Gerber R, Weyl OLF, Sures B, Wepener V, Smit NJ (2019) First record of Labeo capensis (Smith, 1841) in the Crocodile River (West) system: another successful non-native freshwater fish introduction in South Africa. Afr J Aquat Sci 44(2):177–181. https://doi.org/10.2989/16085914.2019.1616529

Erasmus JH, Wepener V, Nachev M, Zimmermann S, Malherbe W, Sures B, Smit NJ (2020) The role of fish helminth parasites in monitoring metal pollution in aquatic ecosystems: a case study in the world’s most productive platinum mining region. Parasitol Res 119(9):2783–2798. https://doi.org/10.1007/s00436-020-06813-1

Farquharson C, Wepener V, Smit NJ (2016) Acute and chronic effects of acidic pH on four subtropical frog species. Water SA 42(1):52–62. https://doi.org/10.4314/wsa.v42i1.07

Gerber R, Smit NJ, van Vuren JH, Ikenaka Y, Wepener V (2018) Biomarkers in tigerfish (Hydrocynus vittatus) as indicators of metal and organic pollution in ecologically sensitive subtropical rivers. Ecotoxicol Environ Saf 157:307–317. https://doi.org/10.1016/j.ecoenv.2018.03.091

Holmes M, Taylor JC (2015) Diatoms as water quality indicators in the upper reaches of the Great Fish River, Eastern Cape, South Africa. Afr J Aquat Sci 40(4):321–337. https://doi.org/10.2989/16085914.2015.1086722

Kleynhans CJ (1999) The development of a fish index to assess the biological integrity of South African Rivers. Water SA 25:265–278

Kock A, Taylor JC, Malherbe W (2019) Diatom community structure and relationship with water quality in Lake Sibaya, KwaZulu-Natal, South Africa. S Afr J Bot 123:161–169. https://doi.org/10.1016/j.sajb.2019.03.013

Kruger T, Barnhoorn I, Jansen van Vuren J, Bornman R (2013) The use of the urogenital papillae of male feral African sharptooth catfish (Clarias gariepinus) as indicator of exposure to estrogenic chemicals in two polluted dams in an urban nature reserve, Gauteng, South Africa. Ecotoxicol Environ Saf 87:98–107. https://doi.org/10.1016/j.ecoenv.2012.10.004

Leusch FD, Chapman HF, van den Heuvel MR, Tan BL, Gooneratne SR, Tremblay LA (2006) Bioassay-derived androgenic and estrogenic activity in municipal sewage in Australia and New Zealand. Ecotoxicol Environ Saf 65(3):403–411. https://doi.org/10.1016/j.ecoenv.2005.07.020

Makene VW, Pool EJ (2015) The assessment of inflammatory activity and toxicity of treated sewage using RAW 264.7 cells. Water Environ J 29(3):353–359. https://doi.org/10.1111/wej.12127

Makene VW, Tijani JO, Petrik LF, Pool EJ (2016) Evaluation of cytotoxicity and inflammatory activity of wastewater collected from a textile factory before and after treatment by coagulation-flocculation methods. Environ Monit Assess 188(8):1–11. https://doi.org/10.1007/s10661-016-5441-x

Malherbe W, Mahlangu S, Ferreira M, Wepener V (2015) Fish and macroinvertebrate community composition of a floodplain wetland associated with the Harts River, South Africa, in relation to water quality and habitat parameters. Afr J Aquat Sci 40(3):311–317. https://doi.org/10.2989/16085914.2015.1076715

Malherbe W, Wepener V, Van Vuren JHJ (2016) The effect of a large-scale irrigation scheme on the fish community structure and integrity of a subtropical river system in South Africa. Ecol Indic 69:533–539. https://doi.org/10.1016/j.ecolind.2016.05.005

Malherbe W, Van Vuren JH, Wepener V (2018) The application of a macroinvertebrate indicator in afrotropical regions for pesticide pollution. J Toxicol 2018:2581930. https://doi.org/10.1155/2018/2581930

Malherbe CW (2013) Validation and implementation of an ecological risk assessment (ERA) framework for pesticide use in the Vaalharts irrigation scheme. Doctoral thesis, University of Johannesburg (Auckland Park campus), South Africa

Nibamureke UMC, Barnhoorn IEJ, Wagenaar GM (2016) Health assessment of freshwater fish species from Albasini Dam, outside a DDT-sprayed area in Limpopo province, South Africa: a preliminary study. Afr J Aquat Sci 41(3):297–308. https://doi.org/10.2989/16085914.2016.1172198

Oberholster PJ, Botha AM, Cloete TE (2008) Biological and chemical evaluation of sewage water pollution in the Rietvlei nature reserve wetland area, South Africa. Environ Pollut 156(1):184–192. https://doi.org/10.1016/j.envpol.2007.12.028

Patrick SM, Aneck-Hahn NH, Van Wyk S, Van Zijl C, Huma M, De Jager C (2020) Veterinary growth promoters in cattle feedlot runoff: estrogenic activity and potential effects on the rat male reproductive system. Environ Sci Pollut Res 27:13939–13948. https://doi.org/10.1007/s11356-020-07966-3

Pheiffer W (2017) Polycyclic aromatic hydrocarbons (PAHs) in the aquatic ecosystems of Soweto and Lenasia. Doctoral thesis, North-West University (South Africa), Potchefstroom Campus Pool EJ, Magcwebeba TU (2009) The screening of river water for immunotoxicity using an in vitro whole blood culture assay.

Water Air Soil Pollut 200(1):25–31. https://doi.org/10.1007/s11270-008-9890-x

Pool EJ, van Wyk JH, Leslie AJ (2000) Inflammatory activity as an indicator of water quality: the use of human whole blood cultures. J Immunoass 21(4):387–399. https://doi.org/10.1080/01971520009349544

Pool EJ (2008) The estrogenicity of sewage effluent entering the Eerste-Kuils River catchment system. Water Research Commission. Report No 1590/1/08

Powrie L (2016) Determining the (anti) androgenic activity of agricultural pesticides in water systems with a luminescence bioassay. Master’s dissertation, North-West University (South Africa), Potchefstroom Campus

Du Preez H, Slabbert L (2008) Application of selected in vivo and in vitro biological/biochemical tests to investigate the estrogenic activity in source and potable water. Water Institute of Southern Africa (WISA) Biennial Conference 2008

Prinsloo S, Pieters R, Bezuidenhout CC (2013) A cell viability assay to determine the cytotoxic effects of water contaminated by microbes. S Afr J Sci 109(7–8):2–5. <https://doi.org/10.1590/sajs.2013/20120069>

Sara JR, Smit WJ, Erasmus LJC, Ramalepe TP, Mogashoa ME, Raphahlelo ME, Theron J, Luus-Powell WJ (2014) Ecological status of Hout River Dam, Limpopo province, South Africa, using fish condition and health assessment index protocols: a preliminary investigation. Afr J Aquat Sci 39(1):35–43. https://doi.org/10.2989/16085914.2013.848181

Schoeman FR (1979) Diatoms as indicators of water quality in the upper Hennops river (Transvaal, South Africa). J Limnol Soc South Afr 5(2):73–78. https://doi.org/10.1080/03779688.1979.9633188

Shackleton AL, Schoeman DS, Newman BK (2002) Bioassays for coastal water quality: an assessment using the larval development of Haliotis midae L. Water SA 28(4):457–461. https://doi.org/10.4314/wsa.v28i4.4919

Simba H (2017) Optimisation and application of the GH3. TRE. luc reporter gene bioassay to assess thyroid activity in drinking and source water. Master’s dissertation, University of Pretoria

Slabbert JL, Oosthuizen J, Venter EA, Hill E, Du Preez M, Pretorius PJ (1998) Development of guidelines for toxicity bioassaying of drinking and environmental waters in South Africa. Water Research Commision Report No. 358/1/98

Swart JC, Pool EJ (2009) Development of a bio-assay for estrogens using estrogen receptor alpha gene expression by MCF7 cells as biomarker. J Immunoassay Immunochem 30(2):150–165. https://doi.org/10.1080/15321810902782855

Swart JC, Pool EJ, Van Wyk JH (2011) The implementation of a battery of in vivo and in vitro bioassays to assess river water for estrogenic endocrine disrupting chemicals. Ecotoxicol Environ Saf 74(1):138–143. <https://doi.org/10.1016/j.ecoenv.2010.09.006>

Taylor JC, Prygiel J, Vosloo A, De La Rey PA, Van Rensburg L (2007) Can diatom-based pollution indices be used for biomonitoring in South Africa? A case study of the Crocodile West and Marico water management area. Hydrobiologia 592(1):455–464. https://doi.org/10.1007/s10750-007-0788-1

Tekere M, Sibanda T, Maphangwa KW (2016) An assessment of the physicochemical properties and toxicity potential of carwash effluents from professional carwash outlets in Gauteng Province, South Africa. Environ Sci Pollut Res 23(12):11876–11884. https://doi.org/10.1007/s11356-016-6370-5

Truter JC, Van Wyk JH, Oberholster PJ, Botha AM, De Klerk AR (2016) An in vitro and in vivo assessment of endocrine disruptive activity in a major South African river. Water Air Soil Pollut 227(2):54. https://doi.org/10.1007/s11270-016-2748-8

Van Deventer R, Morris CD, Hill TR, Rivers-Moore NA (2021) Use of biological and water quality indices to evaluate conditions of the upper uMngeni Catchment, KwaZulu-Natal, South Africa. Afr J Aquat Sci. https://doi.org/10.2989/16085914.2021.1941743

Van Rensburg GJ, Bervoets L, Smit NJ, Wepener V, Van Vuren J (2020) Biomarker responses in the freshwater shrimp Caridina nilotica as indicators of persistent pollutant exposure. Bull Environ Contam Toxicol 104(2):193–199. https://doi.org/10.1007/s00128-019-02773-0

Van Zijl MC, Aneck-Hahn NH, Swart P, Hayward S, Genthe B, De Jager C (2017) Estrogenic activity, chemical levels and health risk assessment of municipal distribution point water from Pretoria and Cape Town, South Africa. Chemosphere 186:305–313. https://doi.org/10.1016/j.chemosphere.2017.07.130

Wagenaar GM, Barnhoorn IEJ (2018) Health and chemical burdens of fish species from polluted and hyper-eutrophic freshwater ecosystems in South Africa. Afr J Aquat Sci 43(3):271–280. https://doi.org/10.2989/16085914.2018.1490245

Wepener VV, Van Dyk C, Bervoets L, O’brien G, Covaci A, Cloete Y, (2011) An assessment of the influence of multiple stressors on the Vaal River, South Africa. Phys Chem Earth 36(14–15):949–962. https://doi.org/10.1016/j.pce.2011.07.075
